# Central Adiposity Indicators, Cardiometabolic Markers and Dietary Quality Among Educational Workers in Southern Ecuador: A Cross-Sectional Study

**DOI:** 10.3390/nu18203408

**Published:** 2026-10-17

**Authors:** Verónica Cárdenas-Mazón, Rosario Suárez, Freddy Lining Aveiga-Ligua, Evelyn Frias-Toral, Martina Galasso, Vanessa Arévalo, Estefany Vivanco, Lezli Malla, Patricia Paredes, Ludovica Verde

**Affiliations:** 1 School of Nutrition and Dietetics, Health Sciences Department, Universidad Técnica Particular de Loja, Loja 110107, Ecuador; vcarevalo1@utpl.edu.ec (V.A.); ecvivanco3@utpl.edu.ec (E.V.); lpmalla@utpl.edu.ec (L.M.); pfparedes@utpl.edu.ec (P.P.); 2 School of Medicine, Health Sciences Department, Universidad Técnica Particular de Loja, Loja 110107, Ecuador; rsuarez2@utpl.edu.ec; 3 Carrera de Medicina, Universidad Católica de Santiago de Guayaquil, Guayaquil 090615, Ecuador; freddy.aveiga@cu.ucsg.edu.ec; 4 Department de Bioestadística, Instituto Oncológico SOLCA Guayaquil, Avda. Pedro Menéndez Gilbert, Guayaquil 090505, Ecuador; 5 Escuela de Medicina, Universidad Espíritu Santo, Samborondón 092301, Ecuador; efriast@uees.edu.ec; 6 Centro Italiano per la cura e il Benessere del Paziente con Obesità (C.I.B.O), Unità di Endocrinologia, Diabetologia e Andrologia, Dipartimento di Medicina Clinica e Chirurgia, Università degli Studi di Napoli Federico II, 80131 Naples, Italy; martina.galasso@uniroma5.it (M.G.); ludovica.verde@unina.it (L.V.); 7 Department for the Promotion of Human Sciences and Quality of Life, San Raffaele Roma Open University, Via di Val Cannuta 247, 00166 Rome, Italy; 8 Division of Endocrinology, Department of Medicine, The University of Arizona College of Medicine, Tucson, AZ 85724, USA

**Keywords:** waist circumference, waist-to-hip ratio, BIA-estimated VFL, cardiometabolic markers, dietary quality, occupational health, Ecuador

## Abstract

Background/Objectives: Cardiovascular disease (CV) is the leading cause of death in Ecuador, and educational workers have been described as exposed to occupational stress and sedentary behavior, factors that may increase cardiometabolic risk. This study evaluated the concordance among three central adiposity indicators, waist circumference (WC), waist-to-hip ratio (WHR), and bioelectrical impedance analysis (BIA)-estimated visceral fat level (VFL), and their associations with cardiometabolic markers and dietary quality (DQ) in educational workers in Loja, Ecuador. Methods: Cross-sectional study of 101 workers (73 women, 28 men; mean age 37.7 years). Adiposity was classified using sex-specific cut-offs for WC (≥90/≥80 cm), WHR (≥0.90/≥0.85) (men/women) and a BIA-estimated VFL (>9). Fasting lipid profiles, fasting glucose, and DQ were assessed once, with DQ evaluated using the Diet Quality Questionnaire (DQQ). Statistical analyses included Cohen’s kappa; Spearman correlation, and binary logistic regression. Results: Overweight or obesity was present in 65.3% of participants, and 57.4% had all three central adiposity indicators simultaneously elevated. Indicator concordance was substantial (*κ* = 0.625–0.747). Altered cardiometabolic markers were common, notably low HDL-c (49.0%) and impaired fasting glucose (IFG, 32.3%). In exploratory sex-stratified analyses, WHR showed the strongest correlations with triglycerides (*r_s_* = +0.607) and cumulative altered markers (*r_s_* = +0.450). Age ≥ 40 years independently raised odds of IFG (aOR = 5.5; 95% CI: 2.0–15.3) and hypertriglyceridemia (aOR = 3.8; 95% CI: 1.2–11.8). Teaching staff exhibited lower DQ scores than support staff (*p* = 0.041). DQ scores showed no significant association with metabolic markers. Conclusions: Educational workers in this sample present a heavy burden of central adiposity and metabolic alterations. All three indicators showed substantial concordance; WHR displayed the broadest associations, although the small male subgroup requires larger validation. These associations reflect statistical relationships and agreement in classification, not diagnostic accuracy; no single indicator can be recommended as a standalone screening tool on the basis of these data. Older workers showed a higher burden of cardiometabolic alterations, which may warrant closer attention in future preventive research.

## 1. Introduction

With approximately 18.6 million deaths in 2019, cardiovascular disease (CVD) continues to be the leading cause of premature death globally [1]. CVD, namely ischemic heart disease, cerebrovascular disease, and hypertensive disease, was the major cause of death in Ecuador in 2024, accounting for 25.7% of all documented deaths (22,996 deaths). The overall cardiometabolic burden rises to 30.9% of all deaths in the country when diabetes mellitus (4680 deaths) is taken into consideration [2]. Additionally, one in ten adults aged 40–69 years carries a ten-year cardiovascular risk of at least 30%, and the national prevalence of arterial hypertension is 19.8% [3]. At the subnational level, Loja province records high CVD-related mortality, with risk exceeding 19% in men compared with 6.9% in women [4].

The multifactorial etiology of CVD encompasses both modifiable risk factors, including physical inactivity, dyslipidemia, dysglycemia, central adiposity, and unhealthy dietary patterns, and non-modifiable determinants, such as age, sex and family history [5]. Independently of overall body mass index (BMI), excess visceral fat accumulation, that is, central adiposity, is a recognized driver of insulin resistance, dyslipidemia, and systemic inflammation [6]. Three indicators are widely used to quantify central adiposity in clinical and epidemiological settings: waist circumference (WC), waist-to-hip ratio (WHR), and BIA-estimated VFL. Each presents distinct operational characteristics and sex-specific cut-offs [7,8].

Educational workers, including teaching, administrative, and support staff, have been described in the literature as frequently exposed to sustained psychosocial stress, prolonged sedentary behavior, and limited time for health-promoting activities [9,10], factors that may contribute to maladaptive dietary behaviors and unfavorable metabolic profiles. These occupational exposures were not directly assessed in the present study and are cited here as contextual background. Despite this, evidence directly relating central adiposity to cardiometabolic markers and dietary quality in this specific workforce remains scarce, particularly in Andean Ecuador.

Dietary quality assessment in these populations has been facilitated by the DQQ, a brief, validated instrument that captures the diversity of food groups consumed during the preceding day [11]. The DQQ generates a Global Dietary Recommendations (GDR) score and differentiates between protective and unhealthy food group consumption, enabling population-level screening without the burden of full dietary recalls [12,13].

The primary objective of this study was to assess the concordance among WC, WHR, and BIA-estimated VFL as central adiposity screening tools and to examine their cross-sectional associations with a panel of cardiometabolic markers in educational institution workers in Loja, Ecuador. Secondary objectives were to: characterize the cardiometabolic profile and dietary quality by sex, age group and occupational role; and examine sex-stratified associations between adiposity indicators and cardiometabolic markers, including multivariable models. Blood pressure, although a central component of cardiometabolic and cardiovascular risk, was not available in this study; the cardiometabolic profile examined here is therefore restricted to lipid and glycemic markers.

## 2. Materials and Methods

### 2.1. Study Design and Participants

A cross-sectional study in 2026 was conducted in two educational institutions in Loja, Ecuador. All full-time teaching, administrative, and support workers aged ≥18 years were invited to participate. The institutions were selected on the basis of institutional agreement to participate. Exclusion criteria were pregnancy, breastfeeding, or a chronic disease requiring specialized nutritional support. Of 118 workers invited, 101 participated in the study (response rate: 85.6%) (Figure S1). Prior to any data analysis, age was dichotomized at 40 years, a threshold established a priori based on clinical and epidemiological considerations. Since the incidence of cardiometabolic events increases significantly beyond 40 years of age, most validated cardiovascular risk assessment tools, such as the WHO/ISH cardiovascular risk prediction charts and the WHO HEARTS technical package for cardiovascular disease management in primary care, use 40 as the minimum age for cardiovascular risk stratification [14,15]. Therefore, rather than being determined empirically from the current dataset, this category was chosen based on well-established risk-assessment frameworks.

Written informed consent was obtained from all participants. Ethical approval was granted by the Institutional Ethics Committee (code 2026-01-INT-EO-RM-005) in accordance with the Declaration of Helsinki.

### 2.2. Anthropometric and Body Composition Measurements

Height was measured to the nearest 0.1 cm with a calibrated stadiometer (Seca 213, Hamburg, Germany). Body weight, body fat percentage, fat mass, skeletal muscle mass, and BIA-estimated VFL were obtained by multi-frequency segmental BIA (InBody 120, InBody Co., Seoul, Republic of Korea) [16]. Measurements were carried out in accordance with the manufacturer’s standard operating procedures: subjects were evaluated following a minimum 3 h fast, after bladder voiding, and in a state of euhydration. The manufacturer’s estimation algorithm incorporates sex, age, height, and weight; however, the BIA-estimated VFL elevation criterion (>9) is applied uniformly across sexes. BMI was calculated and classified according to WHO criteria [17].

WC was additionally measured manually at the midpoint between the iliac crest and the lower costal margin, following the WHO protocol [18], and classified as elevated if ≥90 cm in men or ≥80 cm in women, in accordance with the International Diabetes Federation (IDF) criteria for South/Central American populations [19]. Elevated WHR was defined as ≥0.90 in men and ≥0.85 in women, per WHO criteria [7,19]. The InBody 120 provides a BIA-estimated VFL as a dimensionless score (range 1–20), a scale calibrated by the manufacturer against the visceral fat area (VFA) measured in cm^2^. A BIA-estimated VFL > 9 (equivalent to a Visceral Fat Area ≥ 100 cm^2^) was classified as elevated, derived solely from the manufacturer’s classification and not from an independent, population-specific validation study, in which levels 1–9 represent the standard range and levels ≥ 10 indicate excess visceral fat [16]. This threshold is applied uniformly across sexes and has not been validated against imaging-based reference methods (CT or MRI) in this population.

### 2.3. Biochemical Measurements

Capillary blood samples were collected after a minimum 10 h overnight fast. Total cholesterol, HDL-cholesterol (HDL-c), LDL cholesterol (LDL-c), triglycerides, and fasting glucose were determined as single determinations (not in duplicate), using the LipidPro™ system (Infopia Co., Ltd., Anyang, Republic of Korea) with its corresponding lipid profile and glucose test strips, in accordance with the manufacturer’s instructions. The factory-encoded, lot-specific calibration that came with every test-strip batch served as the basis for calibration; no additional internal calibration was carried out. Measurements are certified by the Cholesterol Reference Method Laboratory Network (CRMLN) as meeting National Cholesterol Education Program (NCEP) performance criteria for accuracy and precision, within recognized performance limits, according to the manufacturer’s validation and the FDA 510(k) decision summary (K090405) [20,21]. LDL-c was not measured directly but calculated by the LipidPro™ system from total cholesterol, HDL-c, and triglycerides. Missing LDL-c values (*n* = 13) occurred mainly because one of these input measurements was unavailable (9 cases); in the remaining cases the device did not report a value despite available input. Dyslipidemia thresholds followed harmonized criteria: elevated total cholesterol > 200 mg/dL, low HDL < 40 mg/dL in men and <50 mg/dL in women, and hypertriglyceridemia ≥ 150 mg/dL [19,22]. Glucose was also assessed as a single capillary fasting measurement, and according to the FDA clearance (K090405) from the LipidPro™ system [20], the glucose function of this device is intended as an aid in the management of diabetes and is not indicated for the diagnosis of or screening for diabetes. Accordingly, glucose values in this study were categorized for research purposes only, using prespecified thresholds (impaired fasting glucose, IFG: 100–125 mg/dL; values ≥ 126 mg/dL), and do not constitute clinical diagnoses, which would require venous laboratory measurement and confirmation on separate days [23].

An exploratory count of altered biomarkers was constructed by summing the number of individually altered cardiometabolic markers per participant (range 0–4). This measure is not a validated cardiometabolic risk assessment, but rather an exploratory count of altered markers. Its components are not clinically similar; LDL-c was not included in this count because it had the highest proportion of missing values (12.9%, *n* = 88 available) and because, being a calculated rather than a directly measured value, it was considered less suitable for a simple count than the directly measured components. Blood pressure could not be included because it was not measured. As a result, the count is not a validated cardiometabolic risk score but rather an equally weighted exploratory descriptive metric. Sex-specific thresholds were used to indicate low HDL-c, but not for the other components. The score was only utilized for descriptive and exploratory purposes because it only accepts five possible values and several adiposity-count categories have few participants. Each of the following conditions contributed one point when present: low HDL-c, IFG, hypertriglyceridemia and elevated total cholesterol. This composite count was analyzed in a Spearman rank correlation to assess the overall cross-sectional association between each adiposity indicator and the aggregate count of cardiometabolic markers.

### 2.4. Dietary Quality Assessment

Dietary quality was assessed using the DQQ, a brief, validated instrument developed by the Global Diet Quality Project to measure population-level food-group consumption based on foods and beverages consumed during the previous day [24]. The instrument retains a common international structure of food groups while the specific foods listed within each group are adapted to each country through a standardized process, in which 5–12 local key informants select representative foods and appropriate terminology for the national context; this adaptation has been completed for 140 countries [25]. A country-specific version adapted to the Ecuadorian food context was used in the present study, without further modification of the published instrument [26]. The DQQ is designed for population-level dietary assessment rather than individual clinical diagnosis or dietary counselling [27,28].

The instrument was administered prior to the anthropometric and biochemical measurements and was given by qualified interviewers during the same assessment session. Following the standardized DQQ recall methodology, participants reported all foods and drinks they had ingested in the preceding 24 h, generating a GDR score (0–18 food categories), a protective food groups score, and an unhealthy food groups score. Higher GDR scores reflect greater alignment with WHO dietary recommendations [28,29].

The DQQ is a single 24 h recall that describes dietary quality and food-group variety at the population level. It is not designed to capture habitual individual consumption or to function as a thorough dietary assessment for individual-level inference.

### 2.5. Statistical Analysis

Continuous variables are displayed as mean ± standard deviation (SD), for descriptive purposes and to facilitate comparison with prior research; for variables that exhibit significant skewness, the median and interquartile range (IQR) are also provided. The Shapiro–Wilk test was used to assess the distribution of continuous data, along with visual examination of histograms and Q-Q plots, skewness, and the small sample size (*n* = 101). The Mann–Whitney U test for two groups, the Kruskal–Wallis test with Dunn’s post hoc test (Bonferroni-corrected) for three groups, and Spearman rank correlation for bivariate associations were the non-parametric methods used for group comparison and bivariate associations, because a number of variables deviated from normality and the sample size limited the robustness of parametric assumptions. Multivariable analysis used regression models (logistic and linear), described below. Fisher’s exact test or chi-square were used to compare categorical variables. Agreement between the categorical classifications produced by each pair of adiposity indicators was quantified by Cohen’s kappa (κ: 0.41–0.60 moderate; 0.61–0.80 substantial; >0.80 near-perfect) [30]. Kappa was interpreted as a measure of concordance in classification, not as evidence of criterion validity, since no imaging-based reference standard was available. Spearman correlations (r_s_) were calculated in the total sample and in sex-stratified subgroups to examine cross-sectional associations.

Binary logistic regression models were constructed to examine covariates independently associated with three outcomes: low HDL-c, IFG and hypertriglyceridemia. As the primary inferential analysis, each adiposity indicator was entered in a separate multivariable model, adjusted for sex and age group, to avoid the mutual attenuation of coefficients that arises when the three highly concordant indicators are entered simultaneously. As a sensitivity analysis, a combined model including all three adiposity indicators together with sex and age group was also fitted. Multicollinearity in this model was assessed using variance inflation factors (VIF), with values below 5 considered acceptable. Sex and age group (≥40 vs. <40 years) were selected a priori as confounders on the basis of their established associations with both adiposity and cardiometabolic markers, and age was also modeled as a continuous variable as part of a sensitivity analysis, and a quadratic term was examined to evaluate any possible non-linearity.

To assess effect modification by sex, logistic regression models were fitted to each outcome, including the adiposity indicator (modelled continuously), sex, and their interaction term (adiposity indicator × sex); a significant interaction term (*p* < 0.05) was interpreted as evidence of effect modification. To examine whether DQ differed by occupational role independently of sex and age, a multivariable linear regression was fitted with the GDR score as a dependent variable and occupational role, sex, and age as associated factors; occupational role was entered as a categorical variable with teaching staff as the reference category, age was modelled continuously, and model assumptions (linearity, homoscedasticity, and normality of residuals) were verified by inspection of residual plots.

Missing biochemical data were handled by listwise deletion; the number of participants included in each regression model is reported in the corresponding table (*n* = 98 for low HDL-c; *n* = 93 for IFG and hypertriglyceridemia). A participant flow diagram is provided in Figure S1. Model discrimination for the logistic models was assessed by AUC-ROC and Nagelkerke’s pseudo-R^2^. Statistical significance was set at α = 0.05. Analyses were performed in SPSS v26.0 (IBM Corp.) and Python 3.11 (statsmodels 0.14; scipy 1.12).

## 3. Results

### 3.1. Sample Characteristics

A total of 101 workers participated: 73 women (72.3%) and 28 men (27.7%), with a mean age of 37.66 ± 10.48 years (range: 23–64). Teaching staff comprised 71.3% (*n* = 72), administrative personnel 18.8% (*n* = 19) and support/services workers 9.9% (*n* = 10). Overall, 64.4% were younger than 40 years. A statistically significant difference was found in occupational role distribution by sex (*p* = 0.018): men were more frequently in support/services roles (21.4% vs. 5.5%), while women were more often in administrative positions (23.3% vs. 7.1%). Education level did not differ significantly by sex (*p* = 0.235), although postgraduate qualifications were more frequent among men (50.0%) than women (35.6%). Sample characteristics are presented in Table 1. All 101 participants had complete anthropometric, body composition, and dietary data; biochemical data were available for 88–100 participants, depending on the analyte, and 85 participants had a complete biochemical panel, because some participants did not complete the blood draw or had insufficient capillary sample volume (Figure S1, Supplementary Table S1).

### 3.2. Body Composition and Prevalence of Elevated Central Adiposity

Two thirds of participants (65.3%) were persons with overweight (44.6%) or obesity (20.8%). Women showed significantly higher body fat percentage (38.7 ± 6.4% vs. 26.9 ± 7.1%, *p* < 0.001), fat mass (26.2 ± 7.7 kg vs. 20.5 ± 8.4 kg, *p* = 0.001), and BIA-estimated VFL (12.2 ± 4.3 vs. 8.1 ± 4.3, *p* < 0.001); men had greater skeletal muscle mass (30.2 ± 2.8 kg vs. 22.0 ± 2.6 kg, *p* < 0.001). In participants aged ≥40 years, all adiposity parameters were significantly elevated: BMI (28.4 ± 3.7 vs. 26.1 ± 3.6 kg/m^2^, *p* = 0.001), WC (92.4 ± 9.7 vs. 86.4 ± 9.8 cm, *p* = 0.003), WHR (0.92 ± 0.05 vs. 0.90 ± 0.05, *p* = 0.001), and BIA-estimated VFL (13.1 ± 4.9 vs. 10.0 ± 4.3, *p* = 0.001).

Using the study-defined cut-offs, elevated WC was found in 72.3% (*n* = 73/101), elevated WHR in 74.3% (*n* = 75/101), and elevated BIA-estimated VFL in 61.4% (*n* = 62/101). More than half of participants (57.4%, *n* = 58/101) had all three indicators simultaneously elevated; only 21.8% had none elevated. Only BIA-estimated VFL showed a statistically significant sex difference (women 74.0% vs. men 28.6%; *p* < 0.001); differences in WC (*p* = 0.063) and WHR (*p* = 0.094) showed tendencies but did not reach statistical significance. Body composition data are presented in Table 2.

### 3.3. Concordance Among Central Adiposity Indicators

All three indicators showed substantial agreement in classification (κ: WC–WHR = 0.747; WHR–BIA-estimated visceral fat = 0.666; WC–BIA-estimated VFL = 0.625). Overall agreement was highest between WC and WHR (90.1%) and lowest between WC and BIA-estimated VFL (83.2%). It should be noted that these kappa values may be influenced by the high prevalence of elevated adiposity in this sample, and the agreement in classification does not imply that the indicators are interchangeable or equally accurate, since no reference standard was available for comparison. Despite this concordance, BIA-estimated VFL classified markedly fewer men as elevated (28.6%) than WC (57.1%) or WHR (60.7%). This divergence may reflect the characteristics of the sex-neutral BIA-estimated VFL cut-off (>9), which has not been validated against imaging techniques in this population, rather than a true difference in central adiposity. Concordance data are presented in Table 3.

### 3.4. Cardiometabolic Markers

The cardiometabolic profile assessed in this study comprised lipid markers and fasting glucose; blood pressure was not measured and is therefore not included in this characterization. Low HDL-c was the most prevalent cardiometabolic alteration, detected in 49.0% (48/98) of participants, with no statistically significant difference between men (46.4%) and women (50.0%; *p* = 0.753). IFG was present in 32.3% (30/93) of participants, with a markedly higher prevalence in workers aged ≥40 years (52.9%) compared with those under 40 (20.3%; χ^2^ = 9.05, *p* = 0.003). Hypertriglyceridemia was detected in 22.6% (21/93). No participant had a fasting glucose value ≥ 126 mg/dL; given the device’s intended use and the single-measurement design, this does not constitute exclusion of diabetes, which would require confirmatory venous testing. Total cholesterol, triglycerides and LDL-c were all significantly higher in participants aged ≥40 years (all *p* < 0.05). Men had significantly lower HDL-c than women (42.2 ± 11.9 vs. 49.4 ± 10.9 mg/dL, *p* = 0.005), with no significant sex differences for other biochemical markers. Cardiometabolic data are presented in Table 4.

### 3.5. Dietary Quality

The mean GDR score was 10.4 ± 2.5 (range: 3–16). The most frequently consumed food groups were refined cereals (86.1%), eggs (75.2%), starchy vegetables and plantains (70.3%), and poultry (64.4%). Fish and seafood were consumed by only 13.9% of participants, and nuts and seeds by 10.9%. Sweetened hot beverages (57.4%) were the most prevalent unhealthy food group, followed by fried street foods (41.6%). GDR differed significantly across job roles (Kruskal–Wallis H = 6.655, *p* = 0.036): teaching personnel had the lowest GDR score (10.0 ± 2.5), followed by administrative staff (10.7 ± 1.7) and support/services staff (12.2 ± 2.4). Dunn’s post hoc test with Bonferroni correction indicated that this difference was driven by the contrast between teaching and support/services staff (adjusted *p* = 0.041). A multivariable linear regression of GDR score by occupational role adjusted for sex and age was carried out to determine whether these factors were confounding this difference. After correction, the greater GDR of support/services workers compared to teaching staff remained significant (β = +1.81; *p* = 0.029); age was independently linked to GDR (*p* = 0.005), but sex was not (*p* = 0.461). The comparisons between teaching and administrative staff (adjusted *p* = 0.762) and between administrative and support/services staff (adjusted *p* = 0.505) were not statistically significant. Workers over 40 years had significantly higher GDR (11.4 ± 2.1 vs. 9.8 ± 2.6, *p* < 0.001) and lower unhealthy food group scores (1.8 ± 1.4 vs. 2.8 ± 1.8, *p* = 0.005). Men consumed more unhealthy food groups than women (3.2 ± 1.9 vs. 2.2 ± 1.6, *p* = 0.007). GDR did not show statistically significant correlations with any biochemical cardiometabolic marker. Dietary quality scores are presented in Table 5.

### 3.6. Cross-Sectional Associations Between Adiposity Indicators and Cardiometabolic Markers, Sex-Stratified Analysis

Spearman rank correlation only differed between men and women in exploratory sex-stratified analyses. In the total sample, WHR was significantly associated with the count of altered cardiometabolic markers (r_s_ = +0.259, *p* = 0.017), and BIA-estimated VFL with total cholesterol (r_s_ = +0.250, *p* = 0.012). When analyses were stratified by sex, statistically significant associations reached significance among men but not among women. This should not be interpreted as evidence that the associations are absent in women, given the exploratory nature of the analyses and the limited subgroup sizes. WHR showed the largest number of significant correlations in men, with triglycerides (r_s_ = +0.607, *p* < 0.001), the cumulative count of altered cardiometabolic markers (r_s_ = +0.450, *p* = 0.024), and HDL-c inversely (r_s_ = −0.392, *p* = 0.039). WC and BIA-estimated VFL also correlated with triglycerides in men (r_s_ = +0.488, *p* = 0.010 and r_s_ = +0.464, *p* = 0.015). In women, no adiposity indicator reached statistical significance with any biochemical marker. These sex-stratified findings should be considered exploratory, given the limited male subgroup size (*n* = 28). To determine whether these apparent sex differences represented true effect modification, sex × adiposity-indicator interaction terms were tested for each outcome (Supplementary Table S2). Only the WHR × sex interaction for hypertriglyceridemia was statistically significant (*p* = 0.020); the WC × sex and VFL × sex interactions for hypertriglyceridemia showed non-significant trends (*p* = 0.075 and *p* = 0.054, respectively), and no interaction was significant for low HDL-c or IFG (all *p* > 0.34). Sex-stratified results are presented in Table 6. The complete set of tested correlations, including all non-significant associations, is provided in Supplementary Table S3 to allow full assessment of the results and to avoid selective presentation.

### 3.7. Multivariable Logistic Regression

To prevent the mutual attenuation of coefficients that occurs when the three highly concordant indicators are entered concurrently, each adiposity indicator was analyzed as the major inferential analysis in a separate multivariable model adjusted for sex and age group. Age ≥ 40 years was the only covariate substantially linked with IFG and hypertriglyceridemia across all models; none of the three adiposity indicators (WC, WHR, or BIA-estimated VFL) were independently associated with any cardiometabolic outcome in these single-indicator models (Table 7).

A combined model incorporating all three indicators concurrently produced consistent results as a sensitivity analysis, with age > 40 years continuing to be the only significant covariate (Supplementary Table S4). The combined model’s variance inflation factors were all less than 3 (Supplementary Table S4), suggesting that multicollinearity was not the cause of the adiposity markers’ lack of significance.

In a sensitivity analysis that represented age as a continuous variable, each additional year of age was associated with higher odds of IFG (OR = 1.06 per year; 95% CI: 1.01–1.11; *p* = 0.013) and hypertriglyceridemia (OR = 1.07 per year; 95% CI: 1.01–1.13; *p* = 0.014). When a quadratic term (age^2^) was introduced, neither outcome showed any signs of non-linearity (both *p* > 0.79), indicating a fairly linear association across the observed age range and no abrupt threshold effect at 40 years (Supplementary Table S5).

### 3.8. Count of Altered Cardiometabolic Markers by Adiposity Indicators

The cumulative count of adiposity indicators (0–3 elevated indicators) was positively correlated with the number of altered cardiometabolic markers (r_s_ = 0.204, *p* = 0.040). Participants with all three adiposity markers elevated (*n* = 58, 57.4%) had the highest prevalences of low HDL-c (53%), and hypertriglyceridemia (28%), along with the highest mean number of altered cardiometabolic markers (ACM: 1.3 ± 1.2) and proportion with ≥2 altered cardiometabolic markers (36%). Data are shown in Table 8.

## 4. Discussion

This cross-sectional study examined the concordance among three central adiposity screening indicators and their associations with cardiometabolic markers in educational institution workers in southern Ecuador. The findings reveal a high concurrent burden of cardiometabolic alterations in a predominantly young working population: two thirds were people with overweight or obesity, nearly three quarters exceeded at least one adiposity threshold, almost half had low HDL-c, and one in three had IFG. All three indicators showed substantial concordance. In sex-stratified exploratory analyses, WHR showed the broadest cross-sectional associations with cardiometabolic markers in men. Age ≥ 40 years was the only variable independently associated with IFG and hypertriglyceridemia across all models.

Central adiposity was highly prevalent across all three indicators (WC 72.3%, WHR 74.3%, BIA-estimated VFL 61.4%), with 57.4% of participants exceeding all three thresholds. This clustering is consistent with findings in comparable occupational populations in Latin America [1,31]. Concordance between the indicators was substantial (κ: 0.625–0.747), indicating that the three tools classified the same individuals as having elevated central adiposity in a largely consistent manner. This agreement in classification does not, however, imply equivalence in predicting cardiometabolic alterations, as kappa quantifies concordance in categorization rather than criterion validity against a clinical outcome. Additionally, women showed a significantly higher prevalence of elevated BIA-estimated VFL than men, suggesting a higher prevalence of central adiposity according to this parameter. This pattern may appear inconsistent with evidence indicating that men generally carry greater absolute cardiovascular risk [4,32]. However, the higher prevalence of this elevated indicator in women may reflect their adiposity distribution under sex-specific anthropometric cut-offs and should not be interpreted as greater cardiometabolic risk, particularly because the subsequent sex-stratified analyses showed significant associations between adiposity and metabolic markers reached statistical significance among men but not among women. However, it should be noted that formal interaction testing for the WHR–triglyceride association did show statistically significant effect modification by sex (Supplementary Table S2). The remaining sex-specific patterns should be viewed as hypothesis-generating rather than as proof of actual sex differences in the adiposity–cardiometabolic association because, despite their consistent direction, they did not achieve significance in interaction models.

A notable difference emerged in men: BIA-estimated VFL classified only 28.6% as elevated compared with 57.1% for WC and 60.7% for WHR. This divergence may reflect the characteristics of the sex-neutral BIA-estimated VFL cut-off (>9), which has not been validated against imaging techniques in this population, rather than a true underestimation of visceral adiposity; no imaging reference was available to determine the true visceral fat area in this sample. Whether sex-specific BIA-estimated VFL thresholds would improve classification merits evaluation in future research.

Regarding cardiometabolic profile, low HDL-c in 49.0% of participants, equally distributed between sexes, is the most clinically notable finding. This prevalence appears higher than estimates from Ecuadorian national STEPS survey [33] and is particularly concerning given that low HDL-c is independently associated with higher atherogenic risk in the presence of borderline LDL and triglyceride levels [34,35]. This frequency is also higher from some comparable occupational cohorts in Latin America, however, these comparisons should be interpreted with caution as differences in age distribution, laboratory methodology, diagnostic thresholds and population characteristics may influence prevalence estimates. HDL-c is influenced by multiple factors including genetics, physical activity, adiposity, smoking, sex hormones and diet; the current study design does not allow attribution of this finding to any specific cause. Nonetheless, the low consumption of fish (13.9%) and nuts and seeds (10.9%), food groups that have been associated with higher HDL-c in other populations, was notable; however, the cross-sectional design precludes any deduction that the low HDL-c in this cohort was caused by food-group consumption. This association has to be looked into in prospective studies with sufficient power. 

Glucose within ranges from 100 to 125 mg/dL was present in 32.3% of participants, rising to 52.9% in those aged ≥40 years, with no cases of glucose > 126 mg/dl. The strong cross-sectional association between age ≥ 40 years and IFG (adjusted OR of 5.5; 95% CI: 2.0–15.3) is consistent with the well-documented age-related decline in insulin sensitivity and beta-cell function [36,37] and suggests this workforce may represent an accessible pre-disease window. However, given the cross-sectional design, no causal direction can be inferred [38]. We verified the robustness of this association by modeling age continuously because age was dichotomized at 40 years, a category defined a priori on the basis of established cardiovascular risk-assessment frameworks rather than derived from the data; the association persisted and showed no evidence of non-linearity (Supplementary Table S5). This suggests that rather than a physically distinct threshold at age 40, the association represents a continuous age-related gradient in cardiometabolic changes. Therefore, the ≥40-year age group should not be interpreted as proof that metabolic risk increases suddenly at that age, but rather as a practical screening cut-off in line with current risk frameworks.

On the other hand, statistically significant cross-sectional associations between adiposity indicators and cardiometabolic markers were observed almost exclusively in men [39]. WHR showed the largest number of statistically significant correlations with cardiometabolic markers in this subgroup, with triglycerides (r_s_ = +0.607), HDL-c (r_s_ = −0.392) and the cumulative count of altered markers (r_s_ = +0.450). This descriptive observation does not establish diagnostic superiority, which cannot be inferred from correlation analyses. In women, no indicator reached significance with any biochemical marker, despite the high prevalence of elevated adiposity. These findings must be interpreted cautiously: the male subgroup includes only 28 participants, which limits the stability of correlation estimates. Observed associations in men should be treated as exploratory and hypothesis -generating, not as definitive evidence of sex differences.

Biologically, these patterns may be partially explained by the probable predominance of premenopausal women in the sample (mean age 38.6 years), whose fat distribution tends to favor subcutaneous gluteofemoral deposits rather than visceral depots, a pattern that may confer relative metabolic protection [40]. However, as these participants age and transition through menopause, the metabolic implications of their current adiposity burden may become more apparent. Importantly, this biological explanation is therefore offered as a hypothesis rather than an established mechanism, and the absence of menopausal data is a limitation that precludes confirmation of this interpretation.

In the primary analysis, none of the three indicators (WC, WHR, or BIA-estimated VFL) was independently associated with any cardiometabolic outcome in separate models adjusted for sex and age group (Table 7). A combined model including all three indicators gave consistent results, and variance inflation factors were all below 3 (Supplementary Table S4), indicating that multicollinearity did not account for their lack of significance. The most likely explanation is limited statistical power: the models were underpowered to detect modest independent associations, and the confidence intervals were correspondingly wide, particularly for hypertriglyceridemia. These findings should serve as a caution against overinterpreting multivariable estimates from an underpowered sample, rather than evidence that central adiposity is unrelated to cardiometabolic markers in this cohort.

Regarding dietary quality, teaching staff recorded the lowest GDR score (10.0 ± 2.5), below both administrative (10.7) and support/services workers (12.2; *p* = 0.036). After adjusting for age and sex, this occupational-role difference remained, suggesting that it is not solely a reflection of the sex composition of the groups; however, given the small number of support/services personnel (n = 10), this finding should be regarded as exploratory and hypothesis-generating. Although formal education level was documented (Table 1), occupational category does not directly correspond to educational attainment, so the finding is interpreted in terms of occupational role than education per se. While higher educational attainment is generally associated with better quality [41,42,43,44,45,46,47], the lower dietary quality observed among teaching staff more likely reflects structural and occupational constraints of the school environment, reduced and unpredictable meal breaks, high cognitive demand, limited access to healthy food options, and chronic psychosocial stress, than a deficit in nutritional knowledge [48,49,50,51]. Because these factors were not measured, they remain hypotheses requiring prospective evaluation with objective measures of work-related stress and food environment access. Workplace nutrition techniques tailored to the occupational context may be a promising preventative approach, since interventions enhancing obesity awareness have been shown to reduce food cravings and improve metabolic parameters in individuals with overweight and obesity [52].

The significant difference in occupational role distribution by sex, with men overrepresented in support/services roles (21.4% vs. 5.5%) and women in administrative positions (23.3% vs. 7.1%), may partially explain sex differences in dietary quality. Although men were overrepresented in support/services roles, the higher GDR in this category persisted after adjustment for sex, suggesting that the occupational-role difference is not solely attributable to sex composition. These occupational role differences warrant consideration when interpreting dietary quality findings.

Men’s significantly higher unhealthy food group score (3.2 vs. 2.2; *p* = 0.007), paralleled their lower HDL-c levels; however, the cross-sectional design does not allow determination of whether dietary patterns contributed to these lipid alterations.

The lack of substantial association between the GDR score and biochemical indicators should not be taken as proof that, in this cohort, dietary quality has no influence on obesity or cardiometabolic health. The intrinsic intra-individual variability of a single 24 h recall, which restricts its ability to define habitual dietary consumption and reduces relationships with biological outcomes through regression-dilution bias, may largely reflect the cause of this null finding. To accurately investigate diet-biomarker interactions in this cohort, repeated 24 h recalls or multi-day dietary assessments would be necessary [53,54]. This limitation does not invalidate the DQQ findings; rather, it reflects the constraints of measuring dietary exposure with a single-day recall [55].

### Strengths and Limitations

Strengths of this study include simultaneous comparison of three central adiposity indicators, WC and WHR, both applied with sex-specific cut-offs, and BIA-estimated VFL, applied with the manufacture’s sex-neutral threshold, objective body composition assessment by BIA, a comprehensive fasting biochemical panel, and sex-stratified correlation analyses. DQQ is a validated, culturally adaptable dietary screening instrument suitable for population-level assessment. Nonetheless, several limitations must be acknowledged. The cross-sectional design precludes causal inference, and all associations should be interpreted accordingly. The sample size, although sufficient for the descriptive and bivariate analyses, was not based on a priori power calculation and constrained the multivariable modelling. The limited number of men substantially reduces the reliability of sex-stratified estimates, and findings in this subgroup should be considered exploratory. The two institutions were selected by convenience, on the basis of their agreement to participate, rather than through random or representative sampling; this, together with the voluntary participation of workers, may introduce selection bias. In addition, because participants were recruited from only two institutions, potential clustering of individuals within institutions could not be accounted for in the analyses; with only two clusters, multilevel modelling was not feasible, which represents a further limitation. Complete-case analysis was used to address missing biochemical data, which affected 16 out of 101 participants. A comparison of participants with and without missing data (Supplementary Table S6) revealed that missing values were more common among women and younger participants, and those with missing values tended to be younger and have lower waist circumference and BMI. Because this pattern is inconsistent with data missing entirely at random, the complete-case technique may create a slight selection bias that is likely to favor participants with higher adiposity. When interpreting the biochemical and regression results, this limitation should be taken into account. Capillary blood was used to collect biochemical data utilizing a point-of-care system (LipidProTM), which were analyzed as single determinations rather than in duplicate. No independent in-house calibration or assessment of intra-/inter-assay precision was carried out. The biochemical assessment is limited because point-of-care capillary measures may be less accurate than reference laboratory venous procedures, even if the device satisfies National Cholesterol Education Program (NCEP) accuracy criteria. No data were collected on physical activity, smoking, alcohol consumption, medication use, menopausal status, sleep, work schedules or socioeconomic status, all of which may be confounders of cardiometabolic risk, and their absence limits the ability to interpret the observed associations causally. The results represent cardiometabolic markers, not estimated CVD risk. The DQQ captures intake on a single day and may not reflect habitual dietary patterns. BIA-estimated VFL measurements may be affected by the use of a manufacturer-defined cut-off that has not been validated against imaging methods in this population. In addition, although fasting, bladder voiding, and euhydration were standardized, physical activity, alcohol intake, and time of day prior to assessment were not systematically standardized; these factors may influence bioelectrical impedance estimates and represent a further limitation of the VFL measurement. The dichotomization of age decreases statistical information and may misrepresent the significance of a particular cut-point, even though the 40-year cut-off was established a priori on clinical grounds and confirmed by the continuous-age sensitivity analysis (Supplementary Table S5). Finally, findings are specific to educational institution workers in Ecuador and may not be generalizable to other occupational groups or geographical settings.

Furthermore, the multiple exploratory analyses performed, particularly the sex-stratified correlations between adiposity indicators and biochemical markers, increase the risk of Type I error. No formal correction for multiple comparisons was applied given the exploratory nature of these analyses; consequently, the sex-specific associations should be regarded as hypothesis-generating and require confirmation in adequately powered studies.

## 5. Conclusions

Educational institution workers in this sample in southern Ecuador show a high concurrent burden of central adiposity and low HDL-c and IFG, with over half of participants simultaneously exceeding all three adiposity indicator thresholds (WC, WHR and BIA-estimated VFL). The three indicators showed substantial agreement in classifying participants, suggesting that they identified the same individuals as having elevated central adiposity. However, this concordance does not imply equivalence in diagnostic accuracy or predictive validity. Because no imaging-based reference standard was available and neither sensitivity nor specificity was determined, these results do not establish the comparative validity of the indicators or their suitability as standalone screening tools. Similarly, the sex-stratified correlations observed in men reflect statistical associations rather than demonstrated diagnostic performance. Their relative diagnostic performance should therefore be evaluated against a validated reference method in future studies. In exploratory sex-stratified analyses, WHR with sex-specific cut-offs showed the broadest cross-sectional associations with cardiometabolic markers in men, though these findings require confirmation in larger, adequately powered studies. Age ≥ 40 years was the variable most consistently associated with IFG and hypertriglyceridemia; older workers in this sample presented a higher burden of metabolic alterations, which may warrant closer attention in future preventive research in the educational sector. Teaching staff exhibited the lowest dietary quality, whether occupational context-sensitive nutritional strategies could improve dietary quality in this group is a question that warrants prospective evaluation. Dietary quality, as measured by the DQQ, showed no significant associations with adiposity indicators or biochemical markers in this sample, a finding most plausibly attributable to the limitations of single-day dietary assessment rather than to a genuine absence of relationship. Overall, these cross-sectional results identify potentially vulnerable subgroups and produce hypotheses about the relationships between age, cardiometabolic markers, and central adiposity in educational workers; however, they do not establish causal pathways or intervention priorities, which will require prospective, sufficiently powered studies.

## Figures and Tables

**Table 1:** General characteristics of the study sample.

Characteristic	Men (*n* = 28)	Women (*n* = 73)	*p*-Value
Age (years), mean ± SD	35.3 ± 9.9	38.6 ± 10.6	0.120
Age < 40 years, *n* (%)	22 (78.6%)	43 (58.9%)	0.106
Age ≥ 40 years, *n* (%)	6 (21.4%)	30 (41.1%)	
Teaching staff, *n* (%)	20 (71.4%)	52 (71.2%)	0.018 *
Administrative staff, *n* (%)	2 (7.1%)	17 (23.3%)	
Support/services staff, *n* (%)	6 (21.4%)	4 (5.5%)	
Marital status			
Partnered	13 (46.4%)	34 (46.6%)	0.989
Single	15 (53.6%)	39 (53.4%)	
Education level			
High school, *n* (%)	4 (14.3%)	5 (6.8%)	0.235
Graduate, *n* (%)	9 (32.1%)	39 (53.4%)	
Postgraduate, *n* (%)	14 (50%)	26 (35.6%)	
Other	1 (3.6%)	3 (4.1%)	

All continuous variables are expressed as mean ± standard deviation. SD, standard deviation. Significance: * *p* < 0.05.

**Table 2:** Body composition and prevalence of elevated central adiposity by sex.

Variable	Men (*n* = 28)	Women (*n* = 73)	*p*-Value
BMI (kg/m^2^), mean ± SD	26.3 ± 3.2	27.1 ± 4.0	0.184
BMI category, *n* (%)			
Healthy weight [18.5–24.9 kg/m^2^]	10 (35.7%)	25 (34.2%)	0.088
Overweight [25.0–29.9 kg/m^2^]	16 (57.1%)	29 (39.7%)	
Obesity [≥30.0 kg/m^2^]	2 (7.1%)	19 (26.0%)	
Body fat (%), mean ± SD	26.9 ± 7.1	38.7 ± 6.4	<0.001 **
Fat mass (kg)	20.5 ± 8.4	26.2 ± 7.7	0.001 *
Skeletal muscle mass (kg)	30.2 ± 2.8	22.0 ± 2.6	<0.001 **
Waist circumference (cm)	91.9 ± 10.5	87.2 ± 9.7	0.054
Elevated WC, *n* (%)	16/28 (57.1%)	57/73 (78.1%)	0.063
Waist-to-hip ratio	0.90 ± 0.04	0.91 ± 0.05	0.447
Elevated WHR, *n* (%)	17/28 (60.7%)	58/73 (79.5%)	0.094
BIA-estimated VFL	8.1 ± 4.3	12.2 ± 4.4	<0.001 **
Elevated BIA-estimated VFL, *n* (%)	8/28 (28.6%)	54/73 (74.0%)	<0.001 **
All three indicators elevated, *n* (%)	8/28 (28.6%)	50/73 (68.5%)	0.001 *

Values are mean ± SD or *n* (%). * *p* < 0.05, ** *p* < 0.001. WC cut-offs: ≥90 cm in men, ≥80 cm in women. WHR cut-offs: ≥0.90 in men, ≥0.85 in women. BIA-estimated VFL cut-off: >9, applied equally for both sexes. BMI, body mass index; WC, waist circumference; WHR, waist-to-hip ratio; BIA-estimated VFL, visceral fat level. All three indicators elevated: WC, WHR and BIA-estimated VFL.

**Table 3:** Prevalence of elevated central adiposity and agreement among the three indicators.

	WC (*n* = 101)	WHR (*n* = 101)	BIA-Estimated VFL (*n* = 101)	κ
Prevalence—total, *n* (%)	73/101 (72.3%)	75/101 (74.3%)	62/101 (61.4%)	
Prevalence—men, *n* (%)	16/28 (57.1%)	17/28 (60.7%)	8/28 (28.6%)	
Prevalence—women, *n* (%)	57/73 (78.1%)	58/73 (79.5%)	54/73 (74.0%)	
Agreement with WC, %	—	90.1%	83.2%	WC–WHR: 0.747
Agreement with WHR, %	90.1%	—	85.1%	WC–BIA-estimated VFL 0.625
Agreement with BIA-estimated VFL, %	83.2%	85.1%	—	WHR–BIA-estimated VFL: 0.666

κ: Cohen’s kappa coefficient. Substantial agreement: 0.61–0.80; WC, waist circumference; WHR, waist-to-hip ratio; BIA-estimated VFL, visceral fat level.

**Table 4:** Cardiometabolic markers by age group.

Variable	Total	<40 Years (*n* = 65)	≥40 Years (*n* = 36)	*p* a	
Total cholesterol					
Mean ± SD (mg/dL)	160.7 ± 32.7	151.0 ± 27.8 (*n* = 64)	178.1 ± 34.0 (*n* = 36)	0.001 *	*
Elevated (>200 mg/dL), *n* (%)	11/100 (11.0%)	1/64 (1.6%)	10/36 (27.8%)	<0.001 **	* b
HDL cholesterol					
Mean ± SD (mg/dL)	47.3 ± 11.6	46.4 ± 11.1 (*n* = 63)	48.9 ± 12.4 (*n* = 35)	0.419	
Low HDL, *n* (%) c	48/98 (49.0%)	33/63 (52.4%)	15/35 (42.9%)	0.488	b
LDL cholesterol					
Mean ± SD (mg/dL)	92.9 ± 28.1	86.6 ± 24.5 (*n* = 54)	103.0 ± 30.8 (*n* = 34)	0.009 *	*
Elevated LDL (>100 mg/dL), *n* (%)	35/88 (39.8%)	17/54 (31.5%)	18/34 (52.9%)	0.075	b
Triglycerides					
Mean ± SD (mg/dL)	117.8 ± 60.9	106.0 ± 48.8 (*n* = 57)	136.4 ± 73.1 (*n* = 36)	0.048 *	*
Median (IQR)	105.0 (71.0–147.0)				
Elevated (≥150 mg/dL), *n* (%)	21/93 (22.6%)	8/57 (14.0%)	13/36 (36.1%)	0.026 *	* b
Fasting glucose					
Mean ± SD (mg/dL)	95.2 ± 10.5	92.7 ± 10.4 (*n* = 59)	99.7 ± 9.1 (*n* = 34)	0.001 *	*
Normal (<100 mg/dL), *n* (%)	63/93 (67.7%)	47/59 (79.7%)	16/34 (47.1%)	0.003 *	* b
Impaired fasting glucose, *n* (%)	30/93 (32.3%)	12/59 (20.3%)	18/34 (52.9%)	0.003 *	* b
Diabetes mellitus (≥126 mg/dL), *n* (%)	0/93 (0.0%)	0/59 (0.0%)	0/34 (0.0%)	—	

Values are mean ± SD or *n* (%). a *p* value from Mann–Whitney U test (continuous variables). b *p* value from Pearson chi-square test (categorical variables). * *p* < 0.05, ** *p* < 0.001. Denominators vary due to missing values; actual n is shown in parentheses. c Low HDL: <40 mg/dL in men, <50 mg/dL in women. Impaired fasting glucose, impaired fasting glucose (100–125 mg/dL); LDL, low-density lipoprotein; HDL, high-density lipoprotein; SD, standard deviation. Glucose categories are based on single capillary fasting measurements (LipidPro™) classified using prespecified research thresholds and are descriptive only; they do not represent clinical diagnoses.

**Table 5:** DQQ dietary quality scores by sex, age group and job role.

DQQ Score	Total (*n* = 101)	Men (*n* = 28)	Women (*n* = 73)	<40 Years (*n* = 65)	≥40 Years (*n* = 36)
GDR score (0–18)	10.4 ± 2.5	10.1 ± 2.4	10.5 ± 2.5	9.8 ± 2.5	11.4 ± 2.12 *
Protective food groups score	3.8 ± 1.7	4.3 ± 1.9	3.6 ± 1.6	3.6 ± 1.8	4.2 ± 1.4
Unhealthy food groups score	2.4 ± 1.7	3.2 ± 1.9 *	2.2 ± 1.6	2.8 ± 1.8	1.8 ± 1.4 *
**GDR by job role (H = 6.655, *p* = 0.036)**	—	Teaching: 10.0 ± 2.5	Admin.: 10.7 ± 1.7	—	Support: 12.2 ± 2.4

mean ± SD; * *p* < 0.05 vs. reference group (women for sex comparison; <40 years for age comparison). GDR, Global Dietary Recommendations score (range 0–18; higher = greater alignment with dietary recommendations). Kruskal–Wallis for job role differences: H = 6.655, *p* = 0.036. SD, standard deviation. Dunn’s post hoc (Bonferroni-adjusted): teaching vs. support/services *p* = 0.041; teaching vs. administrative *p* = 0.762; administrative vs. support/services *p* = 0.505.

**Table 6:** Correlations between adiposity indicators and cardiometabolic markers, total sample and stratified by sex.

Adiposity Indicator	Cardiometabolic Markers	Total r_s_	*p*	Men r_s_	*p*	Women r_s_	*p*	Pattern
WC	Triglycerides	+0.192	0.065 †	+0.488 *	0.010	+0.091	0.466	Significant in men
WC	N° CCM	+0.181	0.098 †	+0.180	0.390	+0.182	0.165	
WHR	HDL cholesterol	−0.101	0.322	−0.392 *	0.039	−0.036	0.770	Significant in men
WHR	Triglycerides	+0.194	0.063 †	+0.607 *	<0.001	+0.074	0.553	Significant in men
WHR	N° CCM	+0.259 *	0.017	+0.450 *	0.024	+0.165	0.206	Global (↑ men)
BIA-estimated VFL	Total cholesterol	+0.250 *	0.012	+0.328	0.088 †	+0.150	0.209	Global
BIA-estimated VFL	Triglycerides	+0.131	0.211	+0.464 *	0.015	+0.070	0.575	Significant in men
BMI	Triglycerides	+0.206 *	0.048	+0.567 *	0.002	+0.116	0.352	Global (↑ men)
BMI	HDL cholesterol	−0.060	0.557	−0.389 *	0.041	−0.008	0.951	Significant in men
BMI	N° CCM	+0.249 *	0.022	+0.356	0.081	+0.216	0.098 †	Global

Only variable pairs with at least one significant (*p* < 0.05) or near-significant (*p* < 0.10) association are shown. * *p* < 0.05, † *p* < 0.10. This selection is presented for readability only and does not reflect the full set of analyses performed; the complete matrix of tested correlations is provided in Supplementary Table S3. N° CCM: cumulative cardiometabolic markers (range 0–4; sum of low HDL + IFG + elevated triglycerides + elevated total cholesterol). WC, waist circumference; WHR, waist-to-hip ratio; BIA-estimated VFL, visceral fat level; HDL, high-density lipoprotein; IFG, impaired fasting glucose; *↑* = elevated in. Sex-stratified findings in men (*n* = 28) should be interpreted with caution as exploratory.

**Table 7:** Single-indicator models, each adiposity indicator adjusted for sex and age group.

Predictor	Outcome	Crude OR (95% CI)	Adjusted OR (95% CI)	*p* adj.		Model Fit
**Model A—Elevated WC + sex + age group**						
Elevated WC	Low HDL-c	1.3 (0.5–3.1)	1.4 (0.6–3.7)	0.452		AUC = 0.58; *n* = 98
Age ≥ 40 years	Low HDL-c	0.7 (0.3–1.6)	0.6 (0.3–1.5)	0.259		
Elevated WC	IFG	1.1 (0.4–2.9)	0.7 (0.2–2.0)	0.481		AUC = 0.70; *n* = 93
**Age ≥ 40 years**	**IFG**	**4.4 (1.8–11.1)**	**5.4 (2.0–15.1)**	0.001 *	*	
Elevated WC	Hypertrig.	2.8 (0.8–10.5)	2.2 (0.5–9.0)	0.270		AUC = 0.70; *n* = 93
**Age ≥ 40 years**	**Hypertrig.**	**3.5 (1.3–9.5)**	**3.5 (1.2–10.4)**	0.027 *	*	EPV = 7
**Model B—Elevated WHR + sex + age group**						
Elevated WHR	Low HDL-c	1.9 (0.7–4.8)	2.1 (0.8–5.7)	0.143		AUC = 0.61; *n* = 98
Age ≥ 40 years	Low HDL-c	0.7 (0.3–1.6)	0.6 (0.2–1.4)	0.211		
Elevated WHR	IFG	1.5 (0.5–4.2)	1.1 (0.3–3.3)	0.929		AUC = 0.69; *n* = 93
**Age ≥ 40 years**	**IFG**	**4.4 (1.8–11.1)**	**4.8 (1.8–13.0)**	0.002 *	*	
Elevated WHR	Hypertrig.	1.6 (0.5–5.5)	1.3 (0.4–4.8)	0.675		AUC = 0.68; *n* = 93
**Age ≥ 40 years**	**Hypertrig.**	**3.5 (1.3–9.5)**	**3.9 (1.3–11.6)**	0.014 *	*	EPV = 7
**Model C—Elevated BIA-estimated VFL + sex + age group**						
Elevated VFL	Low HDL-c	1.7 (0.8–4.0)	2.0 (0.8–5.3)	0.141		AUC = 0.61; *n* = 98
Age ≥ 40 years	Low HDL-c	0.7 (0.3–1.6)	0.6 (0.2–1.4)	0.223		
Elevated VFL	IFG	1.4 (0.6–3.5)	1.2 (0.4–3.5)	0.742		AUC = 0.69; *n* = 93
**Age ≥ 40 years**	**IFG**	**4.4 (1.8–11.1)**	**4.7 (1.8–12.6)**	0.002 *	*	
Elevated VFL	Hypertrig.	2.3 (0.8–6.9)	2.9 (0.8–10.6)	0.116		AUC = 0.70; *n* = 93
**Age ≥ 40 years**	**Hypertrig.**	**3.5 (1.3–9.5)**	**3.7 (1.3–11.0)**	0.018 *	*	EPV = 7

Each adiposity indicator was entered in a separate multivariable model, adjusted for sex and age group, to avoid the mutual attenuation of coefficients that arises when the three highly concordant indicators are entered together. Only the age ≥ 40 years and adiposity-indicator rows are shown; the sex covariate is included in every model. * *p* < 0.05. OR, odds ratio; CI, confidence interval; IFG, impaired fasting glucose; AUC, area under the ROC curve; EPV, events per variable; WC, waist circumference; WHR, waist-to-hip ratio; VFL, BIA-estimated VFL. Sample sizes differ by outcome owing to missing biochemical values: *n* = 98 (low HDL-c), *n* = 93 (IFG and hypertriglyceridemia).

**Table 8:** Distribution of altered cardiometabolic markers by number of elevated adiposity indicators.

N° Elevated Adiposity Indicators	*n*	Low HDL	IFG	Hypertrig.	Mean N° ACM	≥2 Cardiometabolic Markers
0 indicators	22	41%	23%	14%	0.8	14%
1 indicator	6	17%	50%	17%	1.0	33%
2 indicators	15	47%	20%	7%	0.8	20%
3 indicators (all elevated)	58	53%	33%	28%	1.3	36%

ACM: count of altered cardiometabolic markers (range 0–4; sum of low HDL + IFG + elevated triglycerides + elevated total cholesterol). Spearman correlation between number of elevated adiposity indicators and number of altered cardiometabolic markers: r_s_ = 0.204, *p* = 0.040. Note: The 1-indicator group (*n* = 6) is very small; results should be interpreted with caution. The count of altered cardiometabolic markers is an equally weighted, exploratory descriptive sum of four altered markers (low HDL-c, IFG, elevated triglycerides, elevated total cholesterol; range 0–4). It excludes blood pressure (not measured) and LDL-c, and does not constitute a validated cardiometabolic score.

## Data Availability

The data that support the findings of this study are available from the corresponding author upon reasonable request.

## Supplementary Materials

The following supporting information can be downloaded at https://www.mdpi.com/article/10.3390/nu18203408/s1. Figure S1: Participant flow diagram; Table S1: Available observations per variable; Table S2: Sex × adiposity-indicator interaction tests; Table S3: Complete Spearman correlation matrix; Table S4: Sensitivity analysis: combined multivariable logistic regression model including all three adiposity indicators simultaneously, with multicollinearity diagnostics; Table S5: Age modelled as a continuous variable; Table S6: Comparison of participants with versus without missing biochemical data.

## Abbreviations

The following abbreviations are used in this manuscript:

AUC-ROC	Area under the receiver operating characteristic curve
BIA	Bioelectrical impedance analysis
BMI	Body mass index
CF	Cardiometabolic factor
CI	Confidence interval
CRMLN	Cholesterol Reference Method Laboratory Network
CVD	Cardiovascular disease
DQQ	Diet Quality Questionnaire
GDR	Global Dietary Recommendations score
HDL-c	High-density lipoprotein cholesterol
IDF	International Diabetes Federation
IFG	Impaired fasting glucose
LDL-c	Low-density lipoprotein cholesterol
OR	Odds ratio
R^2^	Nagelkerke pseudo-R^2^
SD	Standard deviation
VFL	Visceral fat level
WC	Waist circumference
WHR	Waist-to-hip ratio
WHO	World Health Organization
