# Supplementary material for: Central Adiposity Indicators, Cardiometabolic Markers and Dietary Quality Among Educational Workers in Southern Ecuador: A Cross-Sectional Study
Source: Nutrients. 2026 Oct 17;18(20):3408. doi: 10.3390/nu18203408 (PMC13708413; doi:10.3390/nu18203408)
Supplement: Supplementary file 1 [file nutrients-18-03408-s001.zip › nutrients-4569184-supplementary.pdf]

Supplementary Material

Central adiposity indicators, cardiometabolic markers and dietary quality among educational institution workers in southern Ecuador: a cross-sectional study

Figure S1. Participant flow diagram

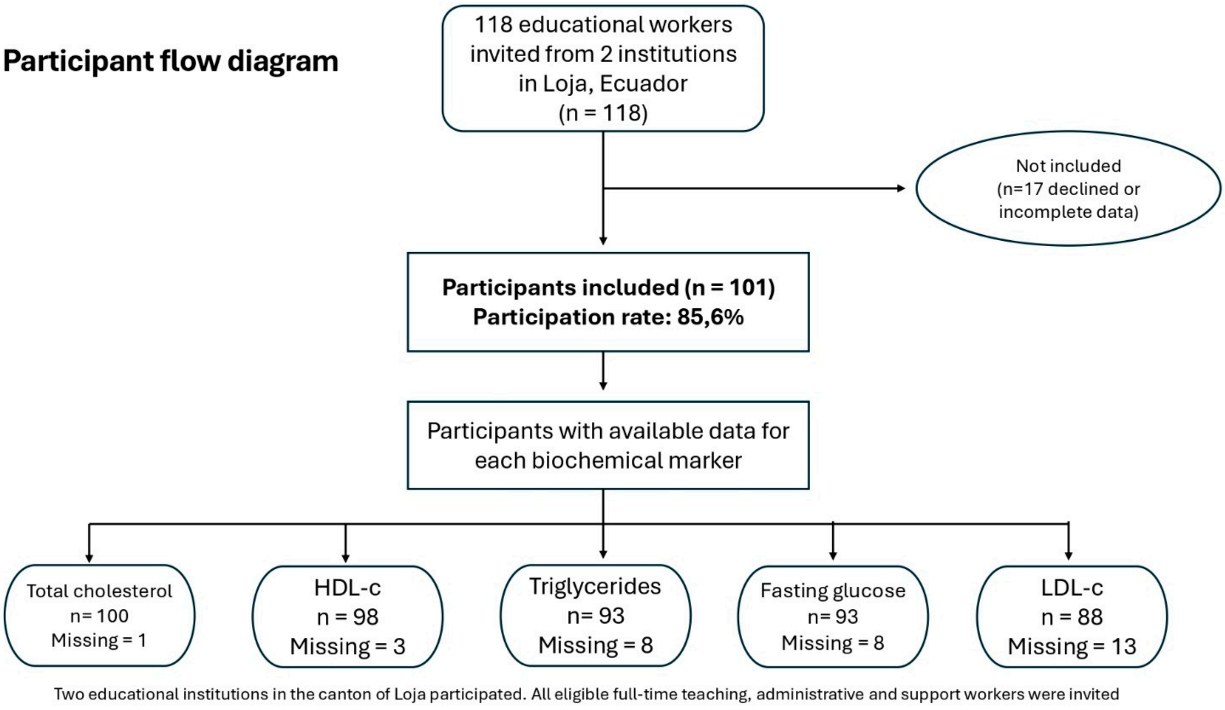

Two educational institutions in the canton of Loja participated. All eligible full-time teaching, administrative and support workers were invited.

Table S1. Available observations per variable

Number of available and missing observations for each major study variable (total N = 101). Anthropometric, body composition and dietary variables were complete; missingness was confined to the biochemical measurements.

Variable	Available n	Missing n	Missing %
Waist circumference	101	0	0.0%
Waist-to-hip ratio	101	0	0.0%
BIA-estimated VFL	101	0	0.0%
BMI	101	0	0.0%
Body fat %	101	0	0.0%
Total cholesterol	100	1	1.0%
HDL-c	98	3	3.0%
LDL-c	88	13	12.9%
Triglycerides	93	8	7.9%
Fasting glucose	93	8	7.9%

GDR score	101	0	0.0%

VFL, BIA-estimated visceral fat level; GDR, Global Dietary Recommendations score. Missing biochemical values arose from insufficient capillary sample volume or non-completion of the blood draw.

Table S2. Sex × adiposity-indicator interaction tests

Tests of effect modification by sex. Each row reports the p-value of the interaction term (adiposity indicator × sex) in a logistic regression model with the outcome, the adiposity indicator (continuous), sex, and their interaction.

Outcome	Adiposity indicator × sex	Interaction p-value
Low HDL-c	WC	0.345
Low HDL-c	WHR	0.406
Low HDL-c	VFL	0.398
IFG	WC	0.383
IFG	WHR	0.820
IFG	VFL	0.722
Hypertriglyceridemia	WC	0.075†
Hypertriglyceridemia	WHR	**0.020***
Hypertriglyceridemia	VFL	0.054†

Only the WHR × sex interaction for hypertriglyceridemia was statistically significant ($p = 0.020$). The WC × sex and VFL × sex interactions for hypertriglyceridemia showed non-significant trends. No interaction was significant for low HDL-c or IFG. These results indicate that, apart from WHR–triglycerides, the apparent sex-specificity of associations reflects differences between subgroup estimates rather than statistically demonstrated effect modification. *$p < 0.05$.

Table S3. Complete Spearman correlation matrix

Full set of Spearman rank correlations between adiposity indicators and cardiometabolic markers, in the total sample and stratified by sex. All tested associations are shown, including non-significant ones, to avoid selective reporting.

Adiposity	Marker	Total rs	p	Men rs	p	Women rs	p
WC	TC	+0.110	0.275	+0.247	0.205	+0.093	0.435
WC	HDL-c	−0.164	0.106	−0.374	0.050†	−0.018	0.885
WC	LDL-c	+0.127	0.238	+0.289	0.153	+0.055	0.673
WC	TG	+0.192	0.065†	+0.488	**0.010***	+0.091	0.466
WC	Glucose	+0.034	0.745	−0.145	0.479	+0.116	0.351
WC	CCM count	+0.181	0.098†	+0.180	0.390	+0.182	0.165
WHR	TC	+0.171	0.088†	+0.346	0.071†	+0.071	0.551
WHR	HDL-c	−0.101	0.322	−0.392	**0.039***	−0.036	0.770
WHR	LDL-c	+0.153	0.154	+0.313	0.120	+0.047	0.715
WHR	TG	+0.194	0.063†	+0.607	**0.001***	+0.074	0.553
WHR	Glucose	+0.141	0.179	+0.110	0.594	+0.158	0.202
WHR	CCM count	+0.259	**0.017***	+0.450	**0.024***	+0.165	0.206
BIA-estimated VFL	TC	+0.250	**0.012***	+0.328	0.088†	+0.150	0.209
BIA-estimated VFL	HDL-c	+0.089	0.382	−0.249	0.201	+0.081	0.505

BIA-estimated VFL	LDL-c	+0.204	0.056†	+0.330	0.100	+0.140	0.279
BIA-estimated VFL	TG	+0.131	0.211	+0.464	**0.015***	+0.070	0.575
BIA-estimated VFL	Glucose	+0.089	0.398	−0.040	0.844	+0.128	0.302
BIA-estimated VFL	CCM count	+0.164	0.132	+0.212	0.310	+0.156	0.235
BMI	TC	+0.219	**0.029***	+0.357	0.062†	+0.147	0.219
BMI	HDL-c	−0.060	0.557	−0.389	**0.041***	−0.008	0.951
BMI	LDL-c	+0.188	0.080†	+0.336	0.093†	+0.125	0.332
BMI	TG	+0.206	**0.048***	+0.567	**0.002***	+0.116	0.352
BMI	Glucose	+0.112	0.286	+0.071	0.731	+0.149	0.228
BMI	CCM count	+0.249	**0.022***	+0.356	0.081†	+0.216	0.098†

rs, Spearman correlation coefficient; TC, total cholesterol; TG, triglycerides; BMI, body mass index; CCM count, count of altered cardiometabolic markers. *$p < 0.05$; †$p < 0.10$. Men: n = 28; women: n = 73 (denominators vary with missing biochemical data). Sex-stratified correlations, particularly in the male subgroup (n = 28), are exploratory and should be interpreted with caution.

Table S4. Sensitivity analysis: combined multivariable logistic regression model including all three adiposity indicators simultaneously, with multicollinearity diagnostics.

**Predictor**	**Outcome**	**Adjusted OR (95% CI)**	**p**		**Model fit**
**Low HDL-c (n = 98)**					
Elevated WC	Low HDL-c	0.56 (0.12–2.68)	0.469		AUC=0.615
Elevated WHR	Low HDL-c	2.24 (0.44–11.51)	0.335		R^2=0.032
Elevated VFL	Low HDL-c	1.77 (0.48–6.51)	0.392		
Male sex	Low HDL-c	1.06 (0.38–2.92)	0.911		
Age ≥ 40 y	Low HDL-c	0.58 (0.24–1.42)	0.235		
**Impaired fasting glucose (IFG) (n = 93)**					
Elevated WC	IFG	0.34 (0.07–1.81)	0.207		AUC=0.705
Elevated WHR	IFG	1.59 (0.25–9.94)	0.621		R^2=0.111
Elevated VFL	IFG	1.72 (0.36–8.13)	0.497		
Male sex	IFG	1.95 (0.60–6.35)	0.269		
**Age ≥ 40 y**	**IFG**	**5.45 (1.95–15.25)**	**0.001***	*	
**Hypertriglyceridemia (n = 93)**					
Elevated WC	Hypertrig.	2.19 (0.26–18.15)	0.468		AUC=0.712
Elevated WHR	Hypertrig.	0.25 (0.03–2.32)	0.222		R^2=0.116
Elevated VFL	Hypertrig.	4.74 (0.63–35.49)	0.130		EPV=4.2
Male sex	Hypertrig.	3.44 (0.89–13.26)	0.073†	†	
**Age ≥ 40 y**	**Hypertrig.**	**3.79 (1.21–11.81)**	**0.022***	*	

Sensitivity analysis: All three adiposity indicators (WC, WHR, VFL) were entered simultaneously, adjusted for sex and age group. Results were consistent with the primary single-indicator analysis (Table 7): no adiposity indicator was independently associated with any outcome, and age ≥ 40 years remained the

sole significant covariate for IFG and hypertriglyceridemia. OR, odds ratio; CI, confidence interval; IFG, impaired fasting glucose; AUC, area under the ROC curve; EPV, events per variable. *p < 0.05; †p < 0.10. n = 98 (low HDL-c), n = 93 (IFG and hypertriglyceridemia).

Multicollinearity diagnostics (variance inflation factors) for the combined model

Predictor	VIF
Elevated WC	2.52
Elevated WHR	2.81
Elevated VFL	2.46
Male sex	1.26
Age ≥ 40 years	1.10

VIF, variance inflation factor. Interpretation: VIF < 5 acceptable; 5–10 moderate; > 10 severe collinearity. All values were below 3, indicating that multicollinearity among the adiposity indicators was not severe and does not explain the non-significance of these indicators in the multivariable models; the wide confidence intervals instead reflect the limited sample size and event counts.

Table S5. Age modelled as a continuous variable

Sensitivity analysis modelling age continuously (multivariable models adjusted for the three adiposity indicators and sex). A quadratic term (age^2) was non-significant for both outcomes (IFG p = 0.797; hypertriglyceridemia p = 0.823), indicating an approximately linear association across the observed age range and no evidence of a threshold effect at 40 years.

Outcome	Age OR (95% CI)	p
IFG (per +1 year)	1.06 (1.01–1.11)	**0.013***
IFG (per +10 years)	1.80 (1.13–2.87)	**0.013***
Hypertriglyceridemia (per +1 year)	1.07 (1.01–1.13)	**0.014***
Hypertriglyceridemia (per +10 years)	1.93 (1.14–3.27)	**0.014***

OR, odds ratio; CI, confidence interval; IFG, impaired fasting glucose. Age modelled as a continuous variable confirms the association observed with the ≥40-year category and suggest it is not an artefact of dichotomization. *p < 0.05.

Table S6. Comparison of participants with versus without missing biochemical data

Comparison of baseline characteristics between participants with complete biochemical data (n = 85) and those with at least one missing biochemical value (n = 16). Participants with missing data tended to be younger and to have lower BMI and waist circumference, though differences did not reach statistical significance. Missingness was more frequent among women (13/73, 17.8%) than men (3/28, 10.7%) and among participants under 40 years (14/65, 21.5%) than those ≥ 40 years (2/36, 5.6%).

Variable	Complete (n=85)	Missing (n=16)	p
Age (years)	38.2 ± 10.9	35.0 ± 7.9	0.322
BMI (kg/m^2)	27.2 ± 3.8	25.3 ± 3.4	0.069†

Waist circumference (cm)	89.3 ± 10.6	84.4 ± 5.3	0.058†
BIA-estimated VFL	11.4 ± 4.8	9.4 ± 3.7	0.138
Male sex, %	29.4%	18.8%	—

Values are mean ± SD or %. p values from Mann–Whitney U test. This pattern is not fully consistent with data missing completely at random; complete-case analysis may introduce a modest selection bias towards participants with higher adiposity, which is acknowledged as a limitation. †$p < 0.10$.
